# Supplementary material for: Enhanced light absorption for solid-state brown carbon from wildfires due to organic and water coatings
Source: Nat Commun. 2024 Nov 28;15:10326. doi: 10.1038/s41467-024-54506-5 (PMC11604999; doi:10.1038/s41467-024-54506-5)
Supplement: Supplementary file 1 — Supplementary Information [file 41467_2024_54506_MOESM1_ESM.pdf]

Supplementary Information for Enhanced light absorption for solid-state brown carbon from wildfires due to organic and water coatings

Zezhen Cheng[1], Manish Shrivastava[2], Amna Ijaz[1,3], Daniel Veghte[1,4], Gregory W. Vandergrift[1], Kuo-Pin Tseng[1,5], Nurun Nahar Lata[1], Will Kew[1], Kaitlyn Suski[2,6], Johannes Weis[7], Gourihar Kulkarni[2], Larry K. Berg[2], Jerome D. Fast[2], Libor Kovarik[1], Lynn R. Mazzoleni[3], Alla Zelenyuk[2], Swarup China[1,*]

[1] Environmental Molecular Sciences Laboratory, Pacific Northwest National Laboratory, Richland, Washington, 99352, USA
[2] Atmospheric, Climate, & Earth Sciences Division, Pacific Northwest National Laboratory, Richland, Washington, 99352, USA
[3] Department of Chemistry, Michigan Technological University, Houghton, Michigan, USA
[4] The Ohio State University, Columbus, Ohio, 43212, USA
[5] University of Illinois at Urbana-Champaign, Champaign, IL, 61801, USA
[6] Rainmaker Technology Corporation, El Segundo, CA, 90245, USA
[7] Chemical Sciences Division, Lawrence Berkeley National Laboratory, Berkeley, California, USA

*Corresponding author:

Swarup China
E-mail: swarup.china@pnnl.gov

Section S1. Single-particle characterization using miniSPLAT

In addition to the microscopy and bulk mass spectrometry analysis of the aerosol filters, in situ characterization of single-particle size, shape, mass spectra, and volatility were performed using the single-particle mass spectrometer, miniSPLAT.[1,2] Based on the mass spectra of individual aerosol particles, particles were classified into eight classes/types. In addition, the average mass spectra and relative number fractions are shown in Fig S1. The average mass spectra of the eight different classes illustrate the differences between the particle composition. For example, it shows that particles classified in Classes 3 and 4 contain a significantly higher fraction of EC with characteristic mass spectral peaks (m/z = 12, 24, 36) than all other classes, while the composition of particles classified in Classes 2, 5, and 8 are dominated by organic carbon, including higher relative fractions of polycyclic aromatic hydrocarbons (PAHs) with mass spectral peaks corresponding to three- to eight-ring PAHs. Note, however, that all of these particles are spherical, highly viscous (trap semi-volatile PAHs, as will be discussed in a separate publication[2]), and have low volatility.[2]

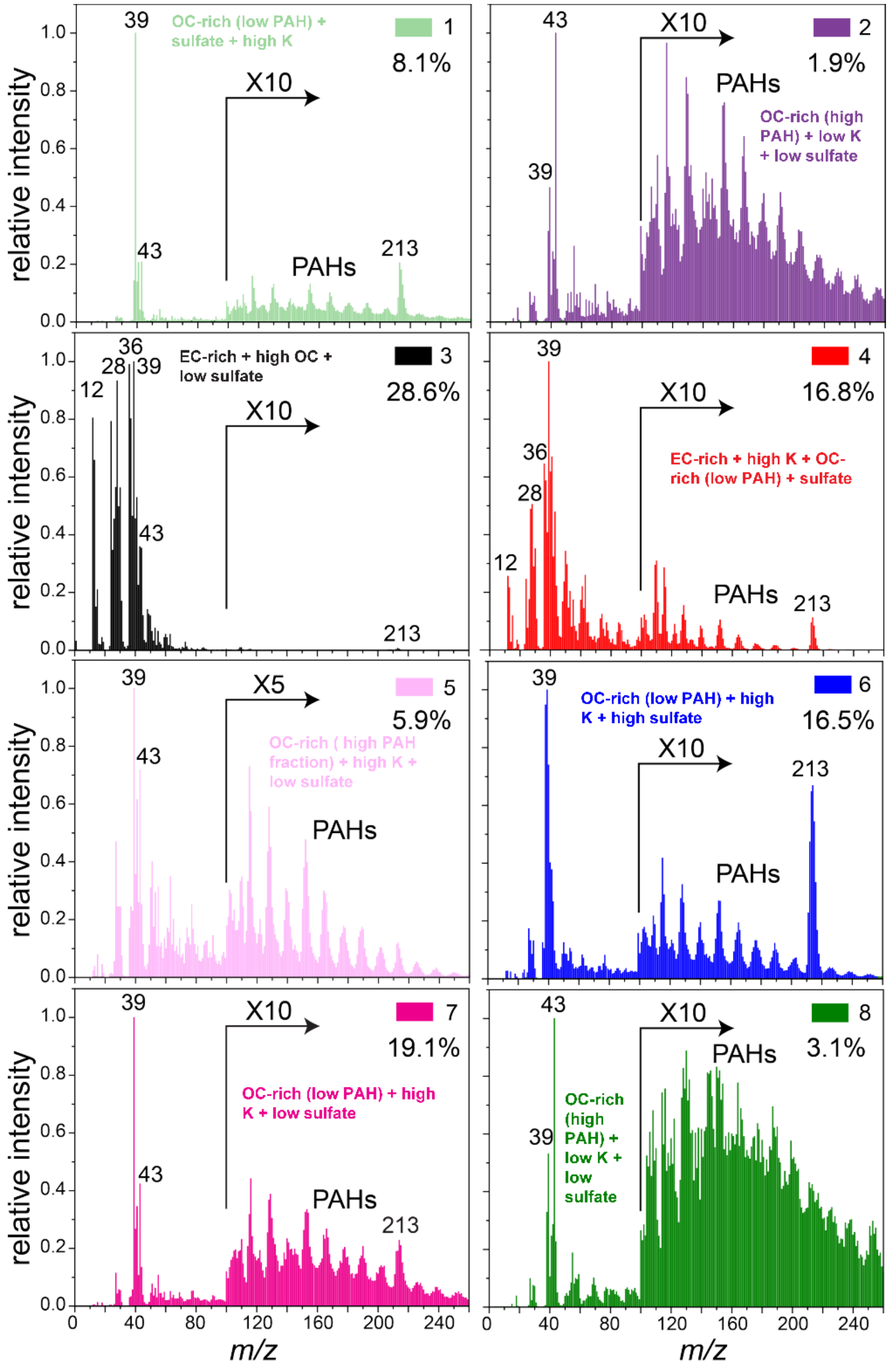

Figure S1. Single particle mass spectra of different particle types. The average miniSPLAT mass spectra and relative number fractions (the number under the colored bars) of eight different classes/types, which are Class 1 (8.1%): OC-rich particles with low PAH fraction, containing sulfate, and high K. Class 2 (1.9%): OC-rich particles with high PAH fraction, low K, and low sulfate. Class 3 (28.6%): EC-rich particles with high OC and low sulfate. Class 4 (16.8%): EC-rich particles with high K, high OC with low PAH fraction, and sulfate. Class 5 (5.9%): OC-rich particles with high PAH fraction, high K, and low sulfate. Class 6 (16.5%): OC-rich particles with low PAH fraction, high K, and high sulfate. Class 7 (19.1%): OC-rich particles with low PAH fraction, high K, and low sulfate. Class 8 (3.1%): OC-rich particles with high PAH fraction, low K, and low sulfate.

Section S2. K-means clustering of O:C ratio

We utilized the *k*-means clustering algorithm to cluster solid S-BrC into low and high O:C elemental ratio groups using CCSEM/EDX and STXM/NEXAFS datasets.[3] For computer-controlled scanning electron microscopy with an energy dispersive X-ray spectrometer (CCSEM/EDX) data, we used the area equivalent diameter, counts per second, and O:C elemental ratio for *k*-means clustering. For scanning transmission X-ray microscopy and near-edge X-ray absorption fine structure (STXM/NEXFAS) data, we used total carbon absorption, area equivalent diameter, and O:C ratio for *k*-means clustering. We used the sqeuclidean distance metric, the maximum number of iterations equal to 10,000, and 10 replicates using new initial cluster centroid positions to ensure we have the best clustering. Results are shown in Fig. S2. The red dots represent particles in cluster 1 with a high O:C ratio, and the blue dots represent particles in cluster 2 with a low O:C ratio and cross-represent the mean of each class.

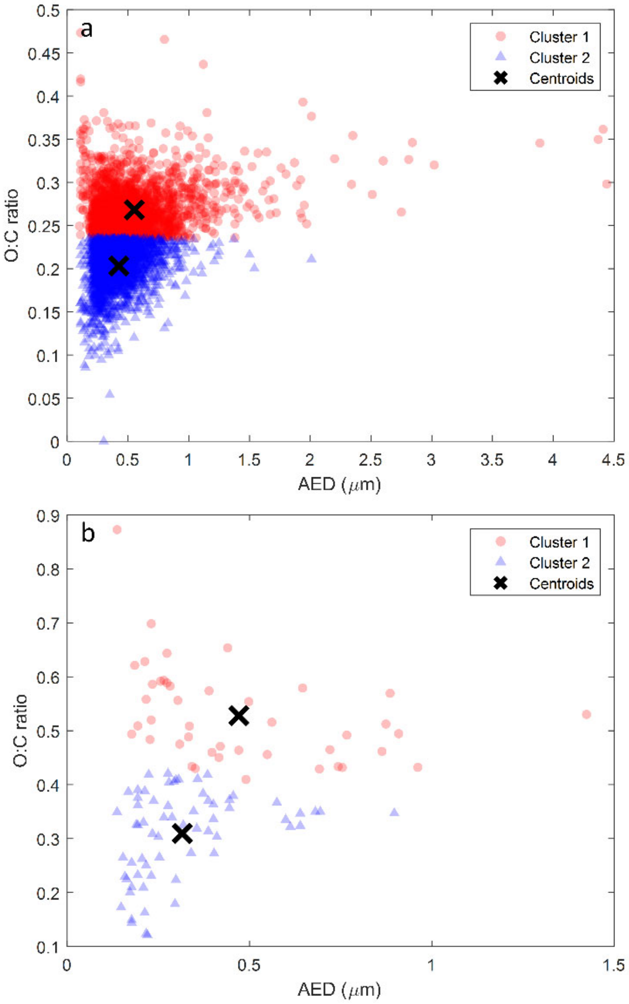

Figure S2. Clustering of individual particles. K-means clustering results for (a) CCSEM/EDX and (b) STXM/NEXAFS.

Section S3. Mie simulation and lensing enhancement calculation

Absorption cross-section (σ_{abs}) and scattering cross-section (σ_{sca}) of solid S-BrC were calculated following the same method introduced by Bond et al., 2006.[4] We used the measured refractive index of solid S-BrC as an import to the Mie theory calculation model. We assume the size distribution of solid S-BrC is a lognormal distribution with the mode varying from 100 to 800 nm with a step size of 50 nm and a geometric standard deviation of 1.1. The model calculates σ_{abs}, σ_{sca}, and the single-scattering albedo (SSA) of the solid S-BrC distribution with a specified mode and geometric standard deviation.

Moreover, we used the core-shell Mie theory model[4] to estimate the lensing enhancement (E_{abs}) at 550 nm of the same solid S-BrC distributions by assuming two different types of coating: (a) clear coating of water due to uptake water in high-humidity environments (Fig. 4b, $RI_{coating} = RI_{water,550} = 1.33+0i$); and (b) brown carbon (BrC) coating due to condensation of organics (Fig. S7, $RI_{coating} = RI_{BrC,550}=1.55+0.01i$).[5] The coating thickness increased from 0 to 2,500 nm with a step size of 10 nm. The E_{abs} of the clear coating is calculated as σ_{abs} of the water-coated solid S-BrC particles ($\sigma_{abs,solid\ S\text{-}BrC,water}$) divided by σ_{abs} of the solid S-BrC cores ($\sigma_{abs,\ solid\ S\text{-}BrC}$). For the BrC coating case, E_{abs} will be more than 4,000 if the BrC coating thickness is significantly larger than the solid S-BrC core diameter, thus dominating the absorption. Therefore, we adopted the parameter, $E_{abs,Remaining}$, defined by Lack and Cappa 2010:[5]

$$E_{\text{abs,Remaining}} = \frac{\sigma_{\text{abs,solid S}-\text{BrC,BrC}} - \sigma_{\text{abs,BrC,BrC}}}{\sigma_{\text{abs,sold S}-\text{BrC,water}}} \quad \text{(S1)}$$

where $\sigma_{abs,\ solid\ S\text{-}BrC,BrC}$ is the σ_{abs} of the BrC-coated solid S-BrC particle, and $\sigma_{abs,BrC,BrC}$ is calculated as σ_{abs} of the BrC particle that is the same size as the coated solid S-BrC particle (core + shell) minus σ_{abs} of the BrC with the size equal to the solid S-BrC core.

Section S4. Direct radiative forcing calculation

The top of the atmosphere direct radiative forcing (TOA-DRF) at a wavelength of 550 nm of solid S-BrC is estimated using the formula provided by Chylek and Wong:[6]

$$\text{DRF} = -\frac{S}{4} \times T^2 \times (1 - C) \times \text{AOD} \times [(1 - a)^2 \times (1 - g) \times \text{SSA} - 4a \times (1 - \text{SSA})], \quad \textit{(S2)}$$

where S is the solar constant (1370 Wm^{-2}), T is the atmospheric transmission (0.79), C is the cloud fraction fixed at a value of 0.6, a is the surface albedo that represents a higher limit for cloud albedo (0.8),[6] AOD is the aerosol optical depth, SSA is the single-scattering albedo obtained from Mie simulations (assume $RI_{clear,550}=1.33$ and $RI_{absorbing,550}=1.55+0.01i$), and g is the asymmetry parameter obtained from Mie simulations. The AOD can be estimated by:[7]

$$\text{AOD} = b_{\text{ext}} \times H, \quad \text{(S3)}$$

where b_{ext} is the extinction coefficient of solid BrC from the Mie simulation, and H is the path length through the aerosol layer.[7] Because the primary purpose of using equation S2 is to estimate the increase of DFR due to clear and absorbing coatings, we assume H is the same for all cases. The lensing enhancement on DRF is defined as:

$$E_{\text{DRF}} = \frac{\text{DRF}_{\text{solid S}-\text{BrC}}}{\text{DRF}_{\text{solid S}-\text{BrC with coating}}}, \quad \text{(S4)}$$

Where $DRF_{solid\ S\text{-}BrC}$ is the DRF of solid S-BrC without a coating, and $DRF_{solid\ S\text{-}BrC\ with\ coating}$ is the DRF of solid S-BrC with either a clear or absorbing coating. The parameters used to estimate E_{DRF} are listed in Table S1.

Table S1. Parameters used to estimate the E_{DRF} at 550 nm for 200 nm solid S-BrC.

Clear coating using RI_{550}=1.33					Absorbing coating using RI_{550}=1.55+0.01i			
Coating thickness (nm)	Extinction coefficient at 550 nm (Mm^{-1})	SSA at 550 nm	g	E_{DRF} at 550 nm	Extinction coefficient at 550 nm (Mm^{-1})	SSA at 550 nm	g	E_{DRF} at 550 nm
100	0.06	0.85	0.57	31.0%	0.11	0.88	0.61	103.9%
200	0.16	0.94	0.70	43.1%	0.33	0.93	0.66	240.0%
300	0.36	0.97	0.77	49.5%	0.71	0.95	0.72	483.8%
400	0.67	0.98	0.80	42.7%	1.17	0.95	0.73	815.9%

Additional Model Details

Table S2. Additional details regarding the regional Weather Research and Forecasting model coupled with chemistry (WRF-Chem 4.2).

**Option**	**Selection**
Boundary Layer Scheme	Yonsei University (YSU)
Land Surface Model	Community Land Model version 4 (CLMv4)
Cloud Microphysics	Morrison Double Moment
Radiation	RRTMG

Table S3. The refractive index used for WRF-Chem 4.2. *n* and *k* were converted to each wavelength using the power-law fitting. The AERONET RI is the average RI during the model simulation period. The data during the smoke peak were missing since the aerosol concentration was too high and beyond the upper detection limit,[48] leading to an underestimate of wildfire aerosol climate effects.

	Wavelength (nm)	*n*	*k*
Scenario 1: average RI values from this study*	300	1.639	0.075
	400	1.664	0.063
	600	1.700	0.054
	999	1.746	0.052
Scenario 2: average RI from AERONET from Aug 11 to 15, 2018	300	1.519	0.009
	400	1.527	0.008
	600	1.539	0.006
	999	1.553	0.004
	300	1.481	0.343

Scenario 3: upper case of RI, *n* from Alexander et al.[8]	400	1.586	0.328
	600	1.671	0.262
	999	1.773	0.218

*: The real part is calculated using the average solid S-BrC real part in the literature.

Table S4. Vertical layers used in WRF-Chem 4.2.

Bin	Mid D_p (nm)
1	1.2207
2	1.9377
3	3.076
4	4.8828
5	7.751
6	12.304
7	19.531
8	31.004
9	49.216
10	78.125
11	124.02
12	196.86
13	312.5
14	496.06
15	787.45
16	1250
17	1984.3
18	3149.8
19	5000
20	7937

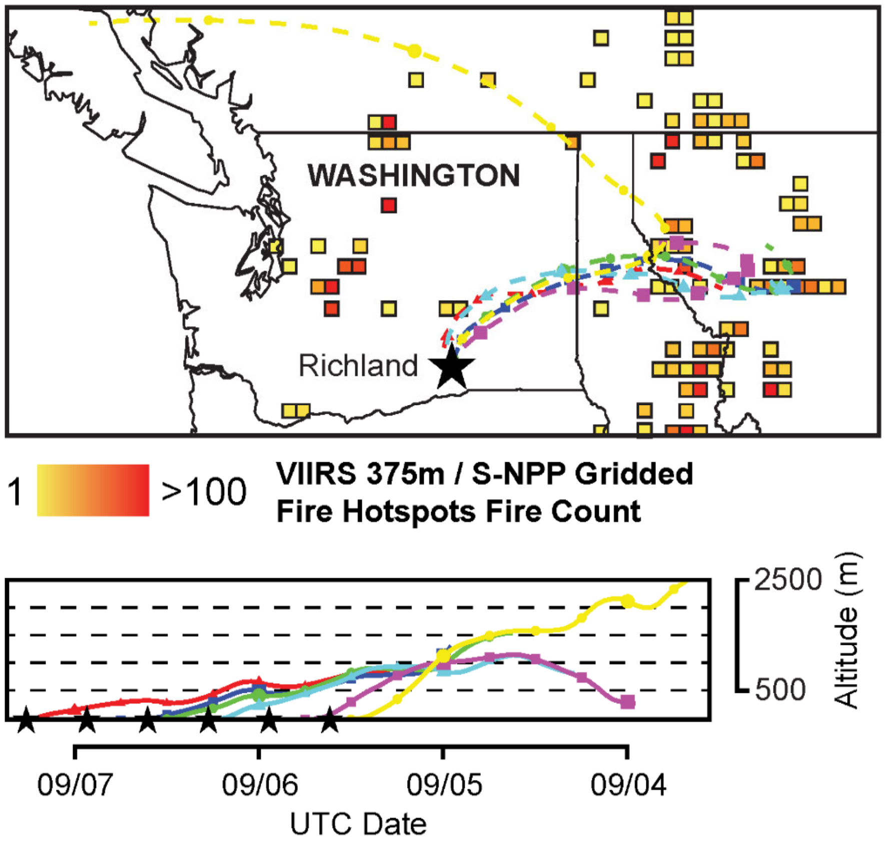

Figure S3. Back trajectory analysis during sampling period. 48-hour back trajectories retrieved from the Hybrid Single-Particle Lagrangian Integrated Trajectory (HYSPLIT)[9] model for the sampling period from September 5, 2017, to September 6, 2017, at 25 m AGL. A new HYSPLIT back trajectory was retrieved every 8 hours, incrementing backward from the point of sample collection (each new trajectory as shown on the map is color-matched on the altitude plot versus time plot). Fire information, which is overlaid on the map and colored according to fire count per grid space, was based on the observation from the Visible Infrared Imaging Radiometer Suite (VIIRS) aboard the Suomi National Polar-orbiting Partnership (S-NPP) satellite.[10] This plot illustrates that most wildfire smoke arriving at the sampling site originated from west Montana State.

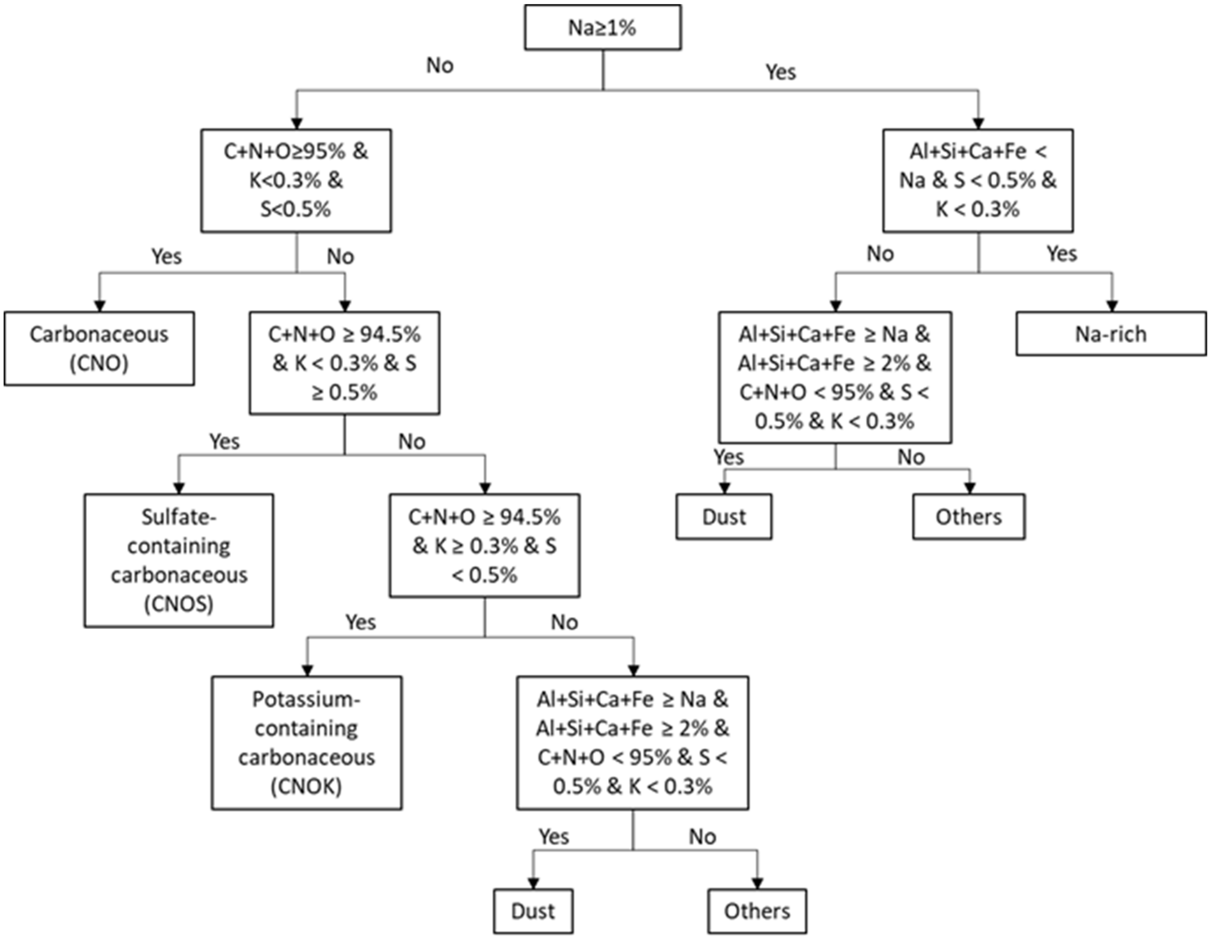

Figure S4. Particle classification scheme. Flow chart for CCSEM-EDX particle classification based on atomic percentage in single particles. The number in the flow chart is the atomic percentage.

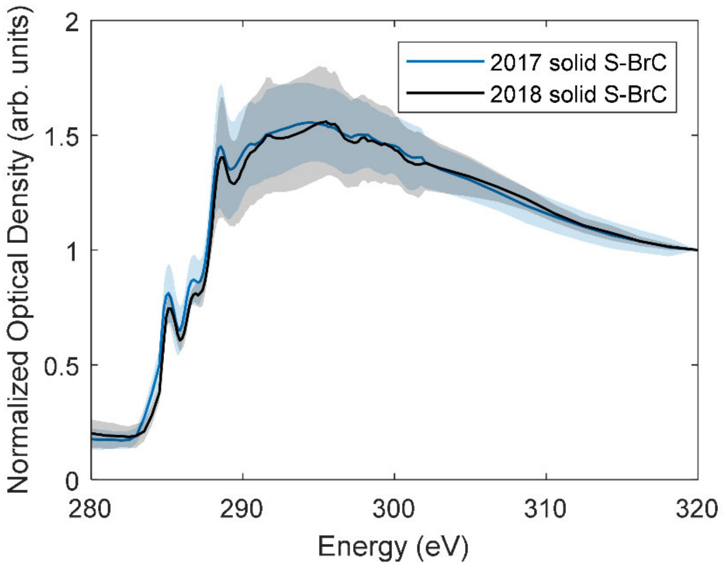

Figure S5. Carbon chemical bonding obtained from X-ray micro-spectroscopy. Averaged

STXM/NEXAFS spectra of individual solid S-BrC particles for 2017 solid S-BrC and 2018 solid S-BrC. The shaded area in (a-c) represents measurement uncertainties as one standard

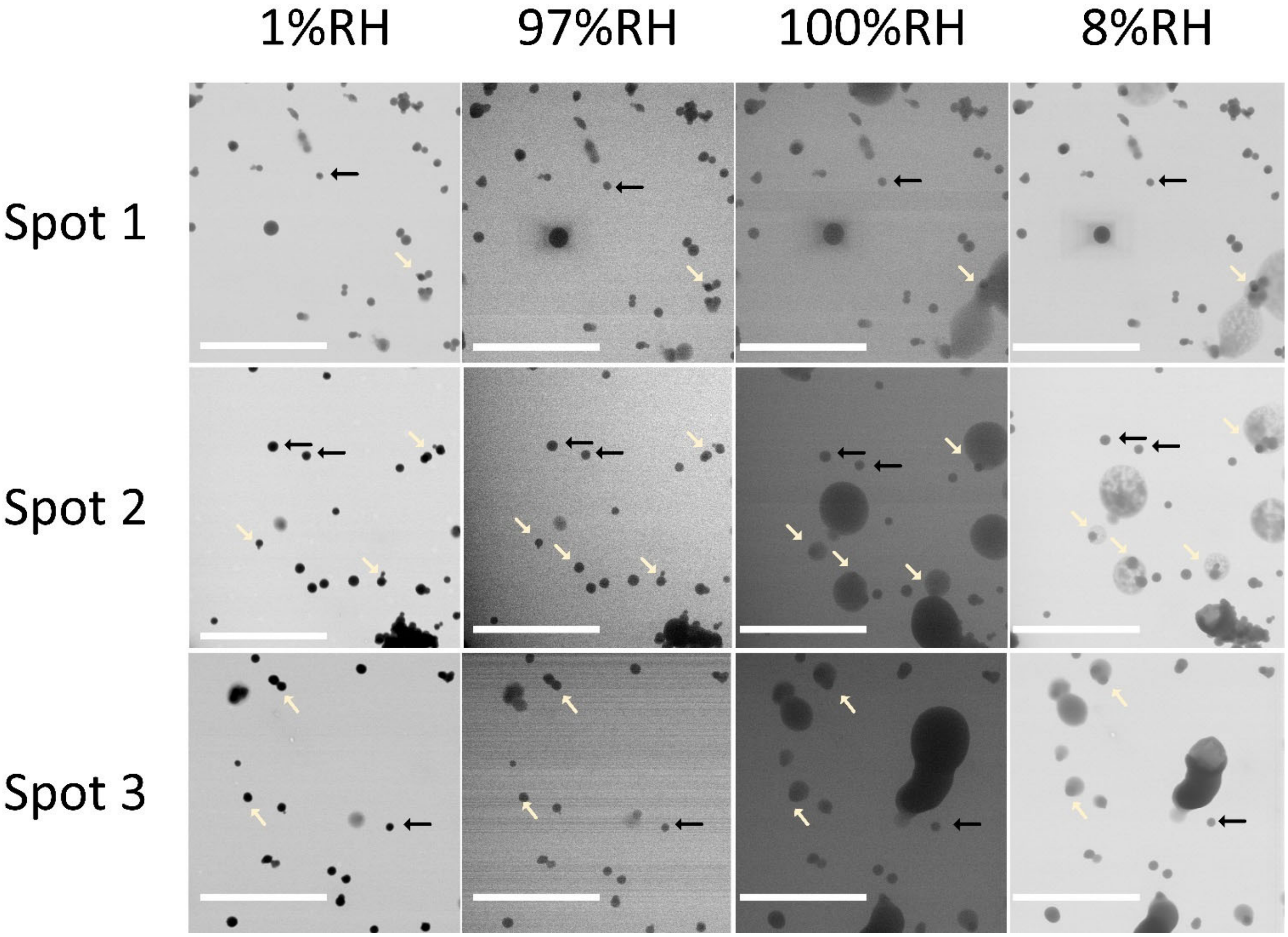

Figure S6. Water uptake of individual particles. Additional environmental scanning electron microscopy images for water uptake experiments from different spots. Scale bars are 5 μm. Black arrows indicate solid S-BrC that do not uptake water, and light yellow arrows indicate solid S-BrC that uptake water and form a core-shell morphology at high RH.

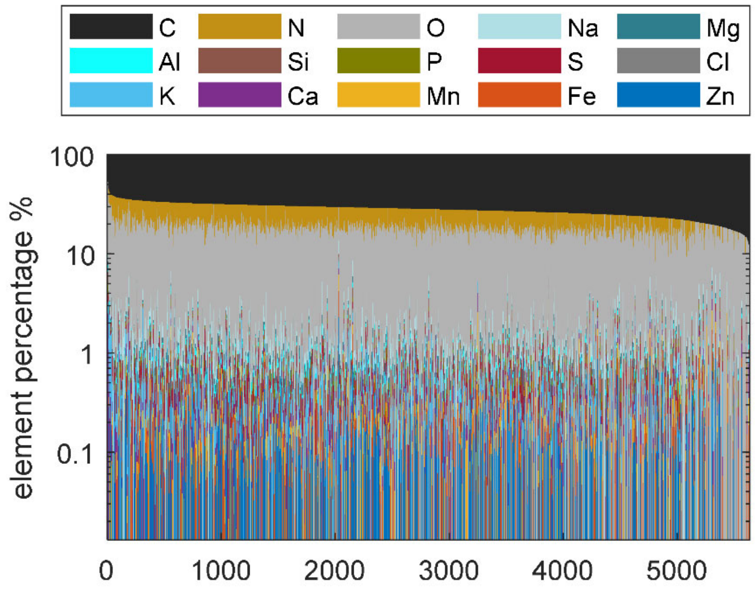

Figure S7. Elemental composition of particles. Elemental percentage of individual particles analyzed by CCSEM-EDX.

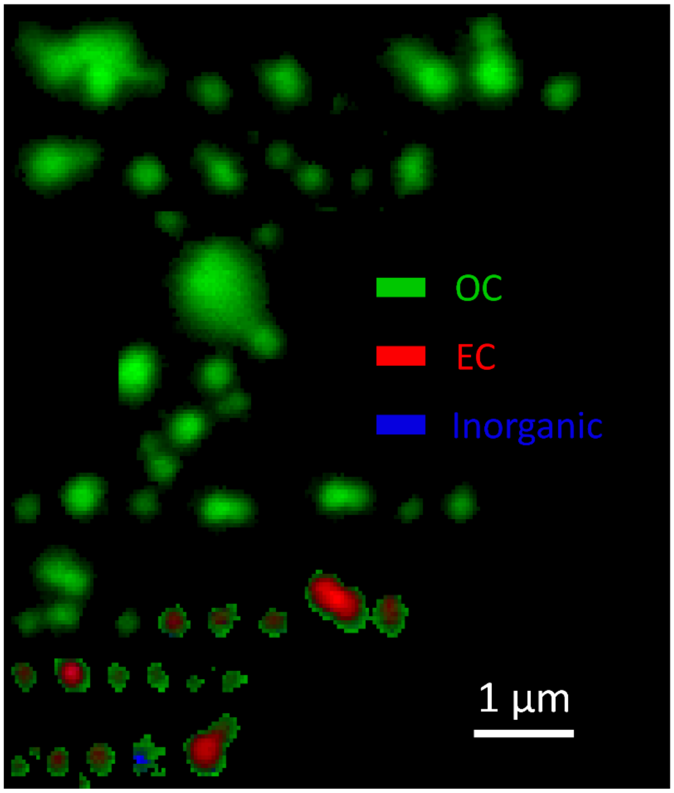

Figure S8. **Chemical imaging of particles obtained from X-ray micro-spectroscopy.** STXM/NEXAFS Carbon speciation maps of solid S-BrC particles.

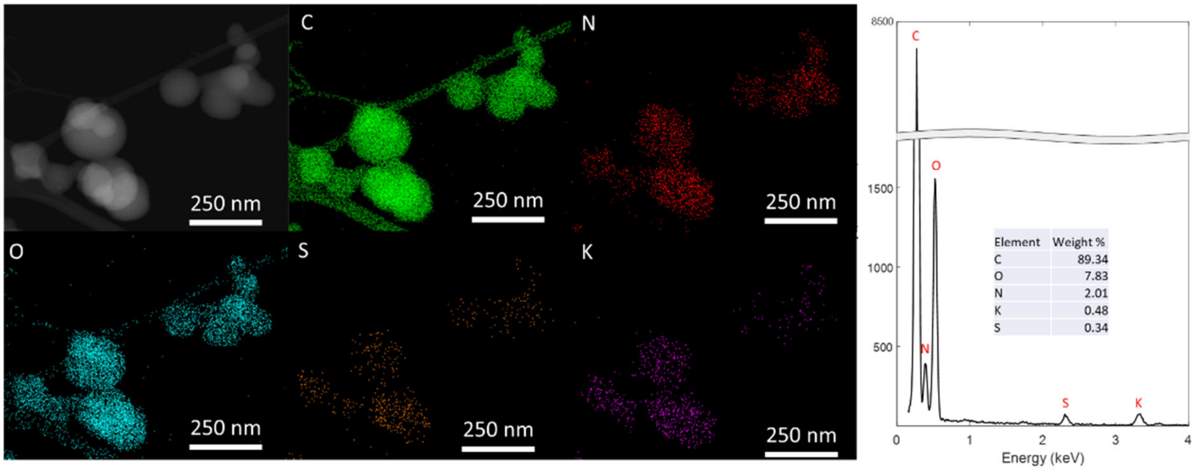

Figure S9. Chemical imaging of particles obtained from electron microscopy. Elemental map of solid S-BrC acquired by Scanning Transmission Electron Microscopy (Thermo Fisher, model Titan 80-300) (STEM)/EDX with energy-dispersive X-ray spectroscopy (Oxford Instruments).

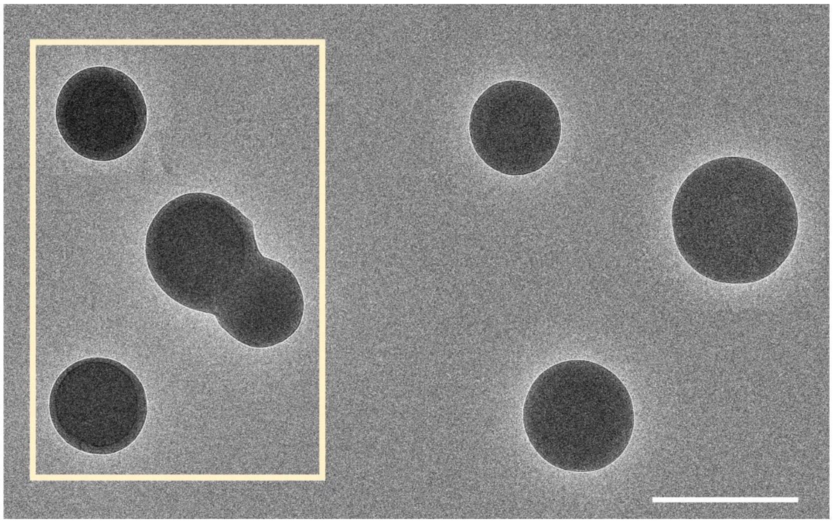

Figure S10. Representative transmission electron microscopy image of solid-state strongly absorptive brown carbon. The solid S-BrC selected by the light-yellow rectangle are solid S-BrC with thin organic coatings and can uptake water. The scale bar is 500 nm.

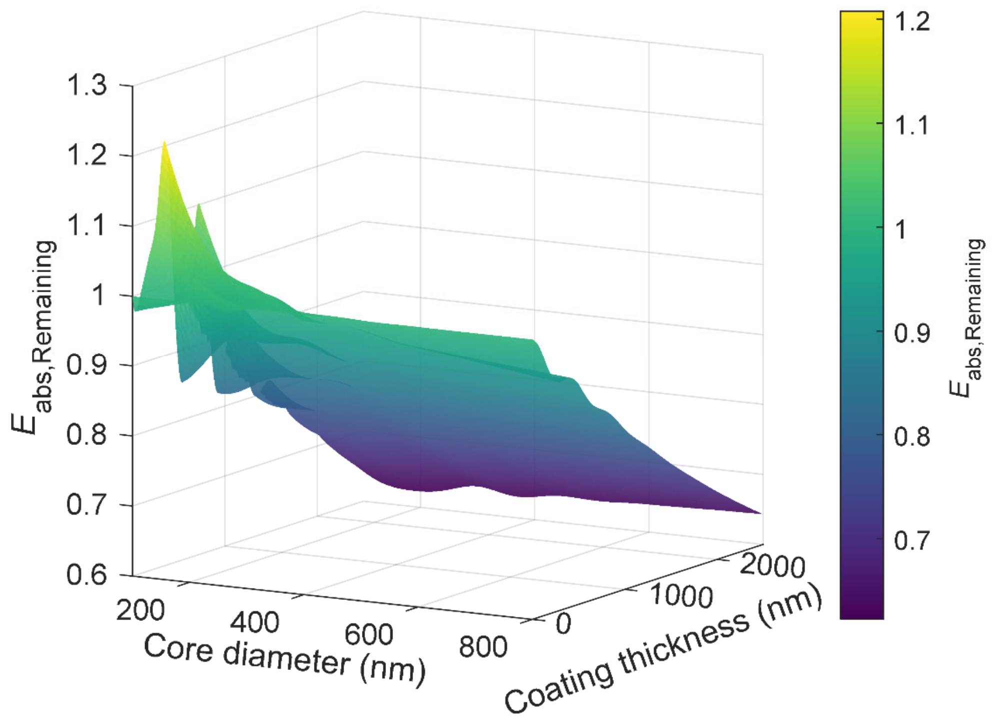

Figure S11. Simulation of absorption enhancement remaining. Calculated absorption enhancement remaining ($E_{abs,Remaining}$) for various solid S-BrC core diameters and BrC coating thicknesses, assuming that the RI of BrC at a wavelength of 550 nm is 1.55+0.01*i*.[5]

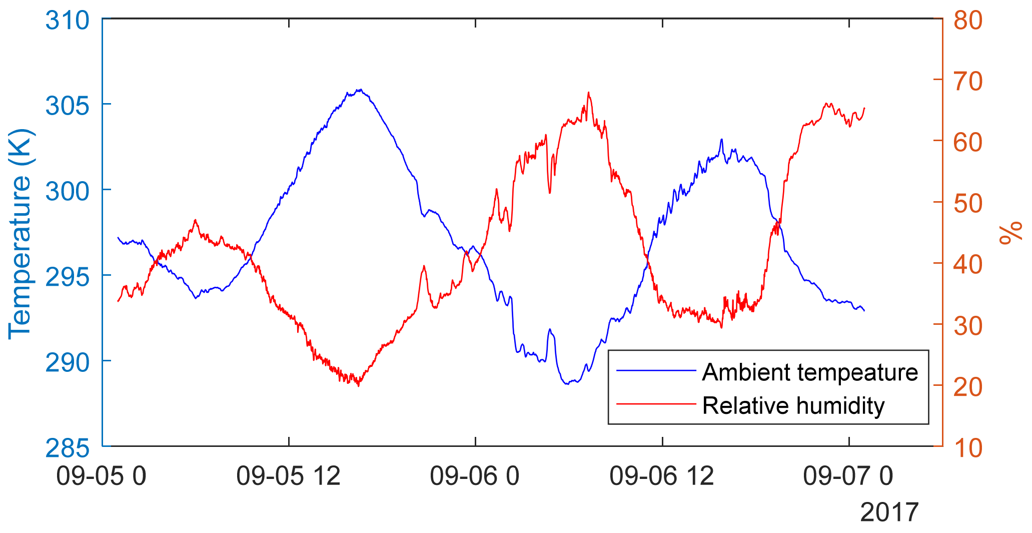

Figure S12. Ambient temperature and relative humidity during the sampling period. Data were reported by the meteorological measurements at the Atmospheric Measurement Laboratory of Pacific Northwest National Laboratory.

References:

1. Zelenyuk, A. *et al.* Airborne Single Particle Mass Spectrometers (SPLAT II & miniSPLAT) and New Software for Data Visualization and Analysis in a Geo-Spatial Context. *J. Am. Soc. Mass Spectrom.* **26**, 257–270 (2015).
2. Brege, M. A., China, S., Schum, S., Zelenyuk, A. & Mazzoleni, L. R. Extreme Molecular Complexity Resulting in a Continuum of Carbonaceous Species in Biomass Burning Tar Balls from Wildfire Smoke. *ACS Earth Sp. Chem.* **5**, 2729–2739 (2021).
3. Hartigan, J. A. & Wong, M. A. Algorithm AS 136: A K-Means Clustering Algorithm. *J. R. Stat. Soc.* **28**, 100–108 (1979).
4. Bond, T. C., Habib, G. & Bergstrom, R. W. Limitations in the enhancement of visible light absorption due to mixing state. *J. Geophys. Res.* **111**, D20211 (2006).
5. Lack, D. A. & Cappa, C. D. Impact of brown and clear carbon on light absorption enhancement, single scatter albedo and absorption wavelength dependence of black carbon. *Atmos. Chem. Phys.* **10**, 4207–4220 (2010).
6. Chylek, P. & Wong, J. Effect of absorbing aerosols on global radiation budget. *Geophys. Res. Lett.* **22**, 929–931 (1995).
7. Pandis, S. and. *Atmospheric Chemistry and Physics: From Air Pollution to Climate Change*. (2006).
8. Alexander, D. T. L., Crozier, P. A. & Anderson, J. R. Brown carbon spheres in East Asian outflow and their optical properties. *Science (80-.).* **321**, 833–836 (2008).
9. Stein, A. F. *et al.* Noaa's hysplit atmospheric transport and dispersion modeling system. *Bull. Amer. Meteor. Soc.* **96**, 2059–2077 (2015).
10. Schroeder, W., Oliva, P., Giglio, L. & Csiszar, I. A. The New VIIRS 375m active fire detection data product: Algorithm description and initial assessment. *Remote Sens. Environ.* **143**, 85–96 (2014).
